# Multimorbidity, Polypharmacy, and Periodontal Disease Burden in a University Dental Setting: A Cross-Sectional Study

**DOI:** 10.3390/biomedicines14102153

**Published:** 2026-09-23

**Authors:** Marco M. Herz, Michael Scharl, Diana Wolff, Johannes Österreicher, Valentin Bartha

**Affiliations:** 1 Department of Conservative Dentistry, University of Tuebingen, Osianderstr. 2-8, 72076 Tuebingen, Germany; johannes.oesterreicher@med.uni-tuebingen.de; 2 Private Practice, Wolfgang-Brumme-Allee 25, 71034 Boeblingen, Germany; 3 Department for Conservative Dentistry, Heidelberg University, Im Neuenheimer Feld 400, 69120 Heidelberg, Germany; diana.wolff@med.uni-heidelberg.de; 4 Department of Cariology, Endodontology and Periodontology, University of Leipzig, 04103 Leipzig, Germany; valentin.bartha@medizin.uni-leipzig.de

**Keywords:** periodontitis, multimorbidity, polypharmacy, systemic disease burden, medication burden, oral–systemic health, cross-sectional study

## Abstract

**Aim**: To investigate multimorbidity and polypharmacy in periodontal patients and determine whether increasing numbers of documented systemic diseases and medications are associated with periodontal disease burden. **Methods**: This cross-sectional study included 116 patients with periodontitis treated in a university dental setting. Systemic conditions and medications were classified according to ICD-10 and ATC systems. Multimorbidity was defined as ≥2 systemic conditions and polypharmacy as ≥5 medications. Associations of the number of documented systemic diagnoses and medications with mean clinical attachment level (CAL), percentage of sites with probing pocket depth (PPD) ≥ 6 mm, and bleeding on probing (BOP) were examined using multivariable linear regression adjusted for age, sex, and current smoking. **Results**: Participants had a mean age of 63.1 ± 11.2 years; 52.6% were female, and 25.9% were current smokers. Multimorbidity was present in 56.9% and polypharmacy in 24.1%. Periodontitis was predominantly advanced (Stage I/II, 13.8%; Stage III, 65.5%; Stage IV, 20.7%). After adjustment, neither the number of documented systemic diagnoses nor the number of medications was significantly associated with mean CAL, percentage of sites with PPD ≥ 6 mm, or BOP (all *p* > 0.20). Sensitivity analyses using categorical definitions of multimorbidity and polypharmacy yielded consistent results. Increasing age, male sex, and current smoking were associated with higher mean CAL, while current smoking was also associated with a higher percentage of sites with PPD ≥ 6 mm. **Conclusions**: Multimorbidity and polypharmacy were common in this periodontal cohort, but increasing numbers of documented systemic diagnoses and medications were not significantly associated with greater periodontal disease burden after adjustment for age, sex, and current smoking. These findings suggest that simple counts of diagnoses or medications may not adequately capture aspects of systemic health relevant to periodontal disease.

## 1. Introduction

Periodontitis is a major chronic inflammatory disease and is increasingly recognized as relevant to general health. Beyond its established role in tooth loss, periodontitis has been associated with a range of systemic conditions, potentially through inflammatory mediators and transient bacteraemia [1]. The strongest and most extensively studied association concerns diabetes mellitus. Periodontitis has been linked to poorer glycaemic control, while diabetes is associated with an increased risk and severity of periodontal disease, supporting a bidirectional relationship [2,3,4]. Periodontal treatment has also been associated with modest reductions in HbA1c levels of approximately 0.4 percentage points [2]. Beyond diabetes, epidemiological associations have been reported with cardiovascular disease, respiratory disease, rheumatoid arthritis, adverse pregnancy outcomes, and kidney disease. Cardiovascular disease represents another extensively studied association: observational studies have reported an increased risk of cardiovascular events among individuals with periodontitis [5], while both a consensus report and a recent umbrella review have supported an association between periodontal and cardiovascular disease, although shared confounders such as smoking and age remain important considerations [6,7].

Several potentially overlapping mechanisms may contribute to these associations. Periodontal inflammation and transient bacteraemia may contribute to systemic inflammatory burden, whereas systemic diseases and unfavorable metabolic conditions may in turn modify periodontal susceptibility through metabolic, inflammatory, and immune mechanisms [4,8,9]. At the same time, periodontal and systemic diseases share major determinants, including age, smoking, socioeconomic factors, diet, and other lifestyle-related factors [10,11,12]. Consequently, the frequent coexistence of periodontitis and systemic disease does not necessarily imply an independent relationship between periodontal severity and systemic disease burden [8,13,14].

Medication use adds another layer of complexity to the relationship between systemic and periodontal health. Several drug classes may directly affect oral and periodontal tissues. Phenytoin, calcium channel blockers, and cyclosporine A are well-established causes of drug-induced gingival overgrowth [15,16], while antidepressants, diuretics, and antihypertensive medications have been associated with xerostomia, particularly in older patients [17,18]. Glucocorticoid therapy may also influence periodontal health through immunomodulatory effects, and observational studies have reported associations between several medication classes, including ACE inhibitors, statins, and anticoagulants, and periodontitis [19]. However, such associations are difficult to interpret independently of the underlying systemic conditions for which these medications are prescribed.

Despite extensive research on individual systemic diseases and medications, considerably less is known about whether the cumulative burden of systemic disease and medication use is associated with periodontal disease burden. Multimorbidity and polypharmacy are particularly relevant in older patient populations, in whom periodontitis and systemic conditions frequently coexist. However, the coexistence of these conditions does not necessarily imply that a greater number of systemic diseases or medications is independently associated with greater periodontal inflammation or destruction. This distinction is clinically relevant because multimorbidity and polypharmacy are increasingly common, whereas their relationship with periodontal disease severity and clinical manifestations remains insufficiently characterized.

This cross-sectional study therefore aimed to characterize multimorbidity and polypharmacy in a university dental cohort with comprehensive periodontal assessment and to investigate whether increasing numbers of documented systemic diagnoses and medications were associated with periodontal disease burden. Specifically, we examined whether increasing systemic disease and medication burden were associated with periodontal inflammation and destruction and whether these associations persisted after adjustment for major confounders, including age, sex, and current smoking.

## 2. Materials and Methods

### 2.1. Study Design and Setting

This cross-sectional study included patients treated at the Department of Operative Dentistry, University of Tübingen, Germany, between November 2020 and October 2021. Data from the underlying patient cohort have previously been used in publications addressing different research questions [20,21]. The study protocol was approved by the Ethics Committee of the University of Tübingen (reference number 120/2020B02; 2 September 2020). The study was retrospectively registered in the German Clinical Trials Register (DRKS00029310; January 2023). Reporting followed the STROBE guidelines.

Periodontal diagnoses were established by periodontists or senior dentists during the initial examination before assignment to the periodontal treatment course, according to the 2017 World Workshop classification of periodontal and peri-implant diseases and conditions.

### 2.2. Participants

All consecutive patients presenting during the predefined educational period were screened for eligibility (Figure 1). Inclusion required a diagnosis of periodontitis and availability of a complete periodontal examination chart. Exclusion criteria followed institutional policies for treatment within the university dental setting and included contagious infections, severe systemic or life-threatening conditions, and conditions precluding routine dental treatment within the study setting, including HIV, hepatitis C, immunosuppression, leukaemia, dementia, and high-risk anticoagulation. All participants provided written informed consent for the use of anonymized data.

### 2.3. Variables

Demographic and behavioral characteristics, systemic conditions, and medication use were extracted from medical records and standardized admission forms. Demographic variables included age and sex. Tobacco and alcohol use were recorded by patient self-report at admission.

Systemic conditions were coded according to the International Statistical Classification of Diseases and Related Health Problems, 10th Revision (ICD-10), both as individual diagnoses and by ICD-10 chapter. Systemic disease burden was quantified primarily as the total number of documented systemic diagnoses. In addition, the number of affected ICD-10 chapters was recorded, and multimorbidity was defined as the presence of ≥2 documented systemic conditions. For the quantitative measure of systemic disease burden, each separately documented systemic diagnosis was counted as one diagnosis. Diagnoses were counted at the level of the individual documented condition rather than the ICD-10 chapter; consequently, clinically related conditions documented as separate diagnoses were counted separately. The total diagnosis count therefore represents the number of documented individual conditions rather than the number of affected organ systems or ICD-10 chapters.

Patients with fewer than two systemic conditions were retained because the primary analyses examined the number of documented systemic conditions across its full observed range as a quantitative exposure. These patients therefore represented the lower end of the systemic disease-burden distribution and also constituted the reference category in the categorical sensitivity analysis comparing patients with and without multimorbidity.

ICD-10 was used to provide a standardized and reproducible classification of documented systemic conditions and to avoid study-specific post hoc grouping of diseases. ICD-10 chapters were additionally used to describe the distribution of systemic conditions across major diagnostic categories.

Medication use was classified according to the Anatomical Therapeutic Chemical (ATC) classification system to provide a standardized classification of medications according to their principal anatomical and therapeutic groups. Medication burden was quantified primarily as the total number of individual medications. The number of represented ATC groups was additionally recorded, and polypharmacy was defined as the concurrent use of ≥5 medications.

Periodontal status was characterized by the number of teeth, mean probing pocket depth (PPD), mean clinical attachment level (CAL), bleeding on probing (BOP), and the number and proportion of sites with PPD ≥ 4 mm and ≥6 mm. Periodontitis was diagnosed and classified according to the 2017 World Workshop classification of periodontal and peri-implant diseases and conditions [22,23]. A diagnosis of periodontitis required detectable interdental clinical attachment loss (CAL) at ≥2 non-adjacent teeth, or buccal/oral CAL ≥ 3 mm with pocketing > 3 mm at ≥2 teeth, provided that the observed CAL could not be attributed to non-periodontitis-related causes.

Periodontitis was staged from I to IV according to disease severity and complexity and graded from A to C according to the estimated rate of disease progression, in accordance with the 2017 World Workshop framework.

Patients underwent an initial examination by general dentists or periodontists before assignment to the periodontal treatment course. Periodontal examinations within the course were performed by undergraduate dental students under continuous supervision by dentists or periodontists. Periodontal parameters were recorded according to a standardized clinical protocol using fixed examination forms, a predefined examination and tooth sequence, and identical diagnostic instruments throughout the study period. Examinations were performed after supragingival cleaning. No formal inter- or intra-examiner calibration or reliability assessment was performed.

The principal exposures were systemic disease burden, represented by the number of documented systemic diagnoses, and medication burden, represented by the number of individual medications. The principal periodontal outcomes were mean CAL, the percentage of sites with PPD ≥ 6 mm, and BOP, representing complementary aspects of cumulative periodontal tissue destruction, current disease severity, and periodontal inflammation, respectively. Age, sex, and current smoking were considered potential confounders. Multimorbidity and polypharmacy, as defined above, were examined as categorical exposure measures in sensitivity analyses.

### 2.4. Data Sources and Data Quality

Medical history data were obtained using a standardized medical history form completed during the clinical examination, with supplementary questions asked by the examining dentist when clarification was required. Clinical and medical information was entered into a customized Ninox database (version 3.7.14; Ninox Software GmbH, Berlin, Germany), exported to Microsoft Excel (version 16.69.1; Microsoft Corporation, Redmond, WA, USA), and analyzed using JMP (version 19.1; JMP Statistical Discovery LLC, Cary, NC, USA).

Data completeness and consistency were verified by a second reviewer, and discrepancies were resolved by consensus. Missing values were excluded from the respective analyses without imputation.

### 2.5. Study Size

No formal a priori sample size calculation was performed because the study was exploratory and no prespecified primary hypothesis was defined. Instead, a time-based census approach was used, including all eligible patients during the predefined educational period. The final sample size was therefore determined by the number of eligible patients with complete periodontal assessment available during this period.

### 2.6. Quantitative Variables

Continuous variables, including age, number of systemic diagnoses, and number of medications, were analyzed as continuous measures. Binary variables, including sex, current smoking, multimorbidity, polypharmacy, and the presence of selected ICD-10 and ATC categories, were entered as categorical variables. The numbers of systemic diagnoses and medications were retained as continuous exposure measures in the primary analyses to preserve information on increasing disease and medication burden. Conventional categorical definitions of multimorbidity (≥2 documented systemic conditions) and polypharmacy (≥5 medications) were used in sensitivity analyses.

### 2.7. Statistical Methods

Descriptive statistics were reported as means with standard deviations for approximately normally distributed continuous variables and as medians with interquartile ranges for non-normally distributed continuous variables. Categorical variables were summarized as absolute and relative frequencies. Distributional characteristics of continuous variables were assessed using the Anderson–Darling test. Between-group differences were examined using the Mann–Whitney U test for continuous variables and Pearson’s χ^2^ test or Fisher’s exact test, as appropriate, for categorical variables.

Associations between systemic disease burden, medication burden, and periodontal outcomes were examined using multivariable linear regression models. The principal periodontal outcomes were mean clinical attachment level (CAL), the percentage of sites with probing pocket depth (PPD) ≥ 6 mm, and bleeding on probing (BOP).

Separate primary models were fitted for systemic disease burden, represented by the number of documented systemic diagnoses, and medication burden, represented by the number of individual medications. All primary models were adjusted for age, sex, and current smoking.

Age, sex, and current smoking were selected a priori as covariates because they represent major shared determinants of systemic and periodontal disease burden. The number of covariates was deliberately limited in view of the moderate sample size and the exploratory nature of the analyses.

In an additional sensitivity analysis, the model for BOP was further adjusted for the use of antiplatelet and/or anticoagulant medication to assess the potential influence of medications affecting hemostasis on the observed association between sex and BOP.

Sensitivity analyses were performed by replacing the continuous exposure measures with conventional categorical definitions of multimorbidity (≥2 documented systemic conditions) and polypharmacy (≥5 medications). In additional exploratory analyses, selected ICD-10 diagnosis categories and ATC medication groups with sufficient case numbers and clinical relevance were examined separately using the same periodontal outcomes and covariate adjustment.

Results from the regression analyses are reported as unstandardized regression coefficients (β) with 95% confidence intervals (CI) and *p*-values. Model R^2^ and adjusted R^2^ are additionally reported for the primary models. Model fit and regression assumptions were assessed using JMP lack-of-fit tests and inspection of residual-versus-predicted plots.

All statistical tests were two-sided, and *p* < 0.05 was considered statistically significant. Given the exploratory nature of the study, no adjustment for multiple testing was applied; findings from the additional category-specific analyses were therefore considered exploratory and interpreted cautiously. Missing data were handled by complete-case analysis for the respective variables, without imputation.

All statistical analyses were performed using JMP version 19.1 (JMP Statistical Discovery LLC, Cary, NC, USA).

## 3. Results

### 3.1. Study Population and Baseline Characteristics

The analytical cohort comprised 116 patients with a mean age of 63.1 ± 11.2 years; 61 (52.6%) were female, and 30 (25.9%) were current smokers. At least one systemic condition was documented in 85 patients (73.3%). Of the total study population, 19 patients (16.4%) had exactly one systemic condition, while 66 patients (56.9%) had two or more conditions and therefore met the definition of multimorbidity. The remaining 31 patients (26.7%) had no documented systemic condition. The median number of systemic diagnoses was 2 (IQR 0–3.75). Patients used a median of 2 medications (IQR 0–4), and 28 (24.1%) met the definition of polypharmacy (≥5 medications).

Periodontal disease was predominantly advanced, with 16 patients (13.8%) classified as Stage I or II, 76 (65.5%) as Stage III, and 24 (20.7%) as Stage IV. Mean PPD was 3.02 ± 0.58 mm and mean CAL was 3.53 ± 0.86 mm. Median BOP was 21.2% (IQR 9.5–36.3), while the median proportions of sites with PPD ≥ 4 mm and PPD ≥ 6 mm were 24.0% (IQR 12.8–35.9) and 2.5% (IQR 0.7–7.4), respectively (Table 1).

At the level of individual diagnoses, the most frequently documented conditions were hypertension (52/116, 44.8%), type 2 diabetes mellitus (16/116, 13.8%), hypothyroidism (15/116, 12.9%), asthma (11/116, 9.5%), and osteoporosis (9/116, 7.8%). A more detailed overview of the most frequently documented individual systemic diagnoses is provided in Supplementary Table S2.

Regarding medication use, the most frequently documented specific medication classes were antiplatelet agents and lipid-lowering agents (each 18/116, 15.5%), followed by thyroid hormones (16/116, 13.8%), ACE inhibitors (13/116, 11.2%), and diuretics (11/116, 9.5%). Further details on the distribution of medication classes are provided in Supplementary Table S3.

### 3.2. Associations of Systemic Disease and Medication Burden with Periodontal Parameters

In multivariable linear regression analyses adjusted for age, sex, and current smoking, the number of individual diagnoses was not significantly associated with mean CAL (β = 0.026 mm per diagnosis; *p* = 0.441), the percentage of sites with PPD ≥ 6 mm (β = 0.382 percentage points per diagnosis; *p* = 0.237), or BOP (β = 0.285 percentage points per diagnosis; *p* = 0.737). Similarly, the number of individual medications was not significantly associated with mean CAL (β = 0.011 mm per medication; *p* = 0.710), the percentage of sites with PPD ≥ 6 mm (β = 0.222 percentage points per medication; *p* = 0.441), or BOP (β = −0.852 percentage points per medication; *p* = 0.257) (Table 2).

Across both sets of models, higher mean CAL was consistently associated with increasing age, male sex, and current smoking. Current smoking was also associated with a higher percentage of sites with PPD ≥ 6 mm, whereas female sex was associated with higher BOP.

In an additional sensitivity analysis, the BOP model was further adjusted for the use of antiplatelet and anticoagulant medication. Use of these medications was not significantly associated with BOP (antiplatelets: β = 1.54, 95% CI −3.74 to 6.82; *p* = 0.565; anticoagulants: β = 2.69, 95% CI −6.52 to 11.91; *p* = 0.564). Again, the association between sex and BOP was statistically significant (female sex: β = 4.05, 95% CI 0.28 to 7.82; *p* = 0.030; β = 4.14, 95% CI 0.40 to 7.88; *p* = 0.030).

The models explained approximately 16% of the variance in mean CAL, 12–13% in the percentage of sites with PPD ≥ 6 mm, and 7–8% in BOP.

Lack-of-fit tests were non-significant for five of the six primary models. Evidence of lack of fit was observed for the model relating the number of diagnoses to BOP (*p* = 0.006).

Smoking was included in the multivariable analyses as current smoking status rather than as a quantitative measure of smoking exposure. Categorical coefficients are reported according to the parameterization used in JMP and therefore represent the displayed JMP model terms rather than recalculated between-group least-squares mean differences. CAL, clinical attachment level; PPD, probing pocket depth; BOP, bleeding on probing. *n* = 115 for mean CAL and *n* = 116 for sites with PPD ≥ 6 mm and BOP. Asterisks and bold type indicate statistical significance.

### 3.3. Sensitivity Analyses Using Categorical Definitions of Multimorbidity and Polypharmacy

Sensitivity analyses using conventional categorical definitions of multimorbidity (≥2 documented systemic conditions) and polypharmacy (≥5 medications) yielded results consistent with the primary analyses based on continuous disease and medication counts. After adjustment for age, sex, and current smoking, neither multimorbidity nor polypharmacy was significantly associated with mean CAL, the percentage of sites with PPD ≥ 6 mm, or BOP (all *p* > 0.30; Table S1).

### 3.4. Distribution of Comorbidities and Medication Groups

Diseases of the circulatory system (ICD-10 chapter IX) were the most frequently documented systemic disease category, affecting 62 patients (53.4%), followed by endocrine, nutritional and metabolic diseases (chapter IV) in 38 patients (32.8%). Musculoskeletal and connective tissue diseases were documented in 19 patients (16.4%), respiratory diseases in 15 (12.9%), and digestive diseases in 11 (9.5%).

Among medication categories, drugs acting on the cardiovascular system (ATC group C) were most common, used by 50 patients (43.1%), followed by drugs affecting blood and blood-forming organs (group B) in 34 (29.3%) and drugs acting on the alimentary tract and metabolism (group A) in 31 (26.7%). Systemic hormonal preparations, excluding sex hormones and insulins (group H), were used by 24 patients (20.7%), nervous system medications (group N) by 19 (16.4%), and musculoskeletal system medications (group M) by 16 (13.8%) (Table 3).

### 3.5. Exploratory Analyses of Specific Disease and Medication Categories

In exploratory analyses, the two most prevalent and clinically relevant ICD-10 categories—endocrine, nutritional and metabolic diseases (chapter IV) and diseases of the circulatory system (chapter IX)—were examined in separate models adjusted for age, sex, and current smoking. Neither category was significantly associated with mean CAL, the percentage of sites with PPD ≥ 6 mm, or BOP (all *p* ≥ 0.221).

Corresponding analyses of medications acting on the alimentary tract and metabolism (ATC group A) and the cardiovascular system (group C) likewise showed no statistically significant associations with the periodontal outcomes.

The lowest *p*-value was observed for the association between ATC group A medication use and the percentage of sites with PPD ≥ 6 mm (*p* = 0.099). Overall, these exploratory analyses did not identify consistent associations between the selected systemic disease or medication categories and periodontal disease burden (Table 4).

## 4. Discussion

In this cross-sectional study of a predominantly older periodontal population, systemic disease and medication burden were not significantly associated with periodontal disease burden after adjustment for age, sex, and current smoking. Neither the number of documented systemic diagnoses nor the number of medications was significantly associated with mean CAL, the percentage of sites with PPD ≥ 6 mm, or BOP. These findings were consistent in sensitivity analyses using conventional categorical definitions of multimorbidity and polypharmacy and in exploratory analyses of selected ICD-10 disease categories and ATC medication groups. In contrast, established demographic and behavioral factors showed clearer associations with periodontal parameters: increasing age, male sex, and current smoking were associated with higher mean CAL, while current smoking was also associated with a higher percentage of sites with PPD ≥ 6 mm. Female sex was associated with higher BOP. Thus, despite the high prevalence of multimorbidity and polypharmacy in this cohort, our findings did not provide evidence that a greater quantitative burden of systemic disease or medication use was associated with greater periodontal disease burden after adjustment for age, sex, and current smoking.

The association between female sex and higher BOP should be interpreted cautiously, particularly because sex-related differences were not consistent across the periodontal outcomes examined. While male sex was associated with higher mean CAL, female sex was associated with higher BOP, suggesting that sex may relate differently to cumulative periodontal destruction and current gingival inflammatory or bleeding manifestations. A previous study suggested that sex-related differences in periodontal inflammation are heterogeneous and may reflect sex-specific interactions with behavioral and clinical factors rather than a consistent difference in BOP between women and men [24]. Because antiplatelet and anticoagulant medications may influence bleeding-related outcomes, their potential contribution to the observed sex difference in BOP was examined in an additional sensitivity analysis, which yielded no significant associations.

Hormonal factors may also contribute to such differences, as changes in sex hormone levels can influence gingival vascular and inflammatory responses. In particular, menopausal status and hormone therapy may be relevant in an older population; however, these factors were not accounted for in the present analysis. Therefore, the observed association between female sex and BOP should be regarded as an exploratory finding rather than evidence of a sex-specific causal effect.

Epidemiological studies consistently identify age as an important determinant of periodontal disease, with prevalence and cumulative periodontal destruction increasing markedly in older populations [25,26]. Consistent with this, increasing age in our cohort was independently associated with higher mean CAL, although not with the percentage of sites with PPD ≥ 6 mm or BOP. Smoking showed an even more distinct pattern, being independently associated with both mean CAL and the percentage of sites with PPD ≥ 6 mm. These findings emphasize the importance of established demographic and behavioral determinants when examining associations between systemic and periodontal disease burden.

Our findings should be considered in the context of previous studies reporting associations between periodontitis and multimorbidity. In a study of nearly 38,000 dental outpatients, Beukers et al. found that patients with periodontitis were more likely to have two or more chronic conditions and identified distinct comorbidity clusters, including respiratory/immune/gastrointestinal disorders and cardiometabolic disease [27]. Other analyses have likewise reported a higher crude prevalence of multimorbidity among individuals with periodontitis, while associations were substantially attenuated or no longer evident after adjustment for shared determinants such as age and smoking [28]. This pattern is compatible with the common risk factor approach [29], according to which major determinants such as age, smoking, sex, obesity, and other lifestyle-related factors may contribute to both systemic and periodontal disease [30,31]. Accordingly, the coexistence of multimorbidity and periodontitis may partly reflect shared determinants rather than a simple relationship between the number of systemic conditions and periodontal disease severity.

Biological interactions between periodontal and systemic health nevertheless remain plausible. Periodontitis is associated with local inflammation and systemic changes in inflammatory markers, while aging is accompanied by chronic low-grade inflammation and immunological alterations often described as “inflammaging” [32,33,34]. Inflammatory, immune, and metabolic pathways have been proposed to contribute to associations between periodontitis and systemic conditions such as atherosclerotic cardiovascular disease, rheumatoid arthritis, and neurodegenerative disorders [6,35,36,37]. The absence of significant associations with aggregate disease and medication counts should not be interpreted as evidence that systemic health is unrelated to periodontal disease. Rather, our findings indicate that the number of documented diagnoses or medications, considered as quantitative measures of burden, was not independently associated with the periodontal outcomes examined in this cohort. Simple counts assign equal weight to conditions that may differ substantially in severity, duration, inflammatory or metabolic impact, and potential relevance to periodontal tissues. In addition, clinically related conditions were counted separately when they were documented as distinct diagnoses. Thus, the diagnosis count may partly reflect diagnostic granularity rather than entirely independent disease entities.

Similarly, medication counts do not distinguish between drugs with potentially adverse, beneficial, or neutral effects on periodontal health. Alternative approaches, including weighted comorbidity indices such as the Charlson Comorbidity Index, disease-specific measures of severity and duration, patterns of multimorbidity, or markers of metabolic and inflammatory burden, may therefore capture clinically and biologically relevant systemic burden more adequately. Future studies should examine whether such measures reveal associations that are not apparent when systemic disease and medication burden are represented by simple counts.

From a biological perspective, the relationship between systemic and periodontal disease may depend more strongly on the nature and activity of specific conditions than on their number alone. For example, metabolic control in diabetes may be more relevant to periodontal susceptibility than the mere presence of a diabetes diagnosis, while the magnitude of systemic inflammatory activity may similarly be more informative than the number of coexisting conditions. Different systemic diseases may affect periodontal tissues through heterogeneous metabolic, inflammatory, and immune pathways, and these effects are unlikely to be adequately represented by assigning equal weight to each documented diagnosis. Accordingly, the absence of an association with aggregate diagnosis counts does not exclude relationships between periodontal disease burden and specific systemic disease phenotypes or levels of metabolic and inflammatory activity.

Although ICD-10 provided a standardized and reproducible framework for classifying systemic conditions, its diagnostic categories are not specifically designed to reflect biological or pathogenetic relationships with periodontal disease. Diseases within the same ICD-10 chapter may differ substantially in their inflammatory, metabolic, or immunological relevance, while conditions from different chapters may share mechanisms potentially relevant to periodontal health. Thus, alternative approaches incorporating specific disease entities, clinically or pathogenetically defined disease clusters, disease severity and duration, or weighted comorbidity indices may provide a more biologically informative characterization of systemic disease burden in future studies.

The exploratory analyses of specific disease and medication categories were consistent with the primary findings. Neither endocrine, nutritional, and metabolic diseases nor diseases of the circulatory system were significantly associated with mean CAL, the percentage of sites with PPD ≥ 6 mm, or BOP after adjustment for age, sex, and current smoking. Similarly, no statistically significant associations were observed for medications acting on the alimentary tract and metabolism or the cardiovascular system. These findings should be interpreted cautiously because the analyses were exploratory, subgroup sizes were limited, and no adjustment for multiple testing was performed.

A further consideration is that the relationship between systemic disease or medication burden and periodontal outcomes may not be linear. The primary analyses modeled diagnosis and medication counts as continuous exposures, whereas the sensitivity analyses additionally applied the conventional thresholds for multimorbidity (≥2 conditions) and polypharmacy (≥5 medications). The consistency of the findings across these approaches provides some reassurance that the main results were not solely dependent on the continuous exposure definition. However, these predefined thresholds may not correspond to biologically relevant thresholds for periodontal effects, and more complex nonlinear or dose–response relationships cannot be excluded. Larger studies with a broader distribution of systemic disease and medication burden could examine such relationships using more flexible modeling approaches.

Previous studies have reported greater medication use among individuals with periodontitis. A 35-year Swedish cohort found greater long-term use of antidiabetic medications, calcium channel blockers, renin–angiotensin system drugs, statins, and other agents among individuals with periodontitis [38]. A case–control study reported higher use of several medication classes, including ACE inhibitors, antidepressants, lipid-lowering agents, oral antidiabetic medications, and diuretics, among patients with periodontitis [39]. Electronic health record analyses have also identified associations between several medication classes and severe periodontitis [19]. However, medication use is closely linked to age and the underlying systemic conditions for which medications are prescribed, while both multimorbidity and polypharmacy become increasingly prevalent in older populations [40,41]. Our findings therefore suggest that the number of medications alone may have limited value as an indicator of periodontal disease burden. Observed associations between medication use and periodontitis may instead reflect a combination of underlying systemic disease, age, specific pharmacological effects, and other shared determinants.

### 4.1. Limitations and Strengths

The moderate sample size limited the precision of the estimates, particularly in the exploratory analyses of individual ICD-10 and ATC categories. No a priori sample size calculation was performed because of the exploratory nature of the study and the absence of a prespecified primary hypothesis. Consequently, the absence of statistically significant associations should not be interpreted as evidence of no association, as smaller but potentially clinically relevant effects may not have been detected in this cohort. Multiple exploratory analyses were conducted without adjustment for multiple testing. In addition, multimorbidity and polypharmacy were primarily quantified as counts of documented diagnoses and medications and therefore did not capture disease severity, duration, specific combinations, or their biological relevance to periodontal disease. Although categorical sensitivity analyses using conventional definitions of multimorbidity and polypharmacy were consistent with the primary analyses, more complex nonlinear or threshold relationships were not systematically evaluated. Smoking was modeled as a binary current-smoking variable, and differences in smoking intensity and cumulative lifetime exposure were not accounted for. Residual confounding related to smoking exposure therefore cannot be excluded. Residual confounding by factors not included in the multivariable models, including obesity, socioeconomic factors, alcohol consumption, nutrition, and other lifestyle-related factors, cannot be excluded. Sex-specific factors such as menopausal status and hormone therapy were not included in the multivariable models and may also have influenced BOP. Furthermore, BOP is a bounded percentage outcome, and the diagnosis-count model showed evidence of lack of fit; estimates from this model should therefore be interpreted with some caution. Selection bias cannot be excluded because participants were included from a single university dental setting and predominantly represented an older population with advanced periodontitis. The predominance of Stage III and IV periodontitis may also have restricted the range of periodontal disease severity, potentially reducing the ability to detect associations between systemic disease or medication burden and periodontal outcomes across the full spectrum of disease severity. Furthermore, the limited representation of earlier-stage periodontitis restricts the generalizability of the findings to populations with predominantly mild or moderate periodontal disease.

In addition, institutional eligibility criteria excluded patients with certain severe systemic conditions, potentially underrepresenting individuals with the highest systemic disease burden and limiting generalizability. Information bias is also possible because systemic conditions, medication use, and behavioral factors were partly based on information obtained from the medical history and may therefore have been incompletely or inaccurately reported. Although periodontal examinations followed a standardized protocol and were performed under continuous supervision by dentists or periodontists, no formal inter- or intra-examiner calibration or reliability assessment was performed. Examiner-related measurement variability therefore cannot be completely excluded.

Strengths of the study include the inclusion of all eligible patients within a predefined educational period, standardized classification of systemic conditions and medications using ICD-10 and ATC systems, and the availability of detailed clinical periodontal measures. Systemic disease and medication burden were examined using both continuous and conventional categorical definitions, complemented by exploratory analyses of selected disease and medication categories, with consistent adjustment for age, sex, and current smoking.

### 4.2. Practical, Educational, and Research Implications

Although greater systemic disease and medication burden was not significantly associated with greater periodontal disease burden in this cohort, the high prevalence of multimorbidity and polypharmacy underscores the importance of comprehensive medical and medication histories in periodontal care, particularly for treatment planning, potential drug interactions, and interdisciplinary management. In a university teaching setting, these findings also highlight the importance of training dental students to recognize multimorbidity and polypharmacy as common features of periodontal patient care and to integrate systemic health and medication use into clinical assessment and treatment planning. At the same time, the coexistence of periodontitis and multimorbidity should not be assumed to reflect a direct relationship between the quantitative burden of systemic disease and periodontal severity.

From a research perspective, simple counts of diagnoses or medications may not adequately capture the aspects of systemic health that are biologically relevant to periodontal disease. Future studies should therefore consider disease type, severity and duration, specific patterns of multimorbidity and medication use, and measures of metabolic and inflammatory burden. Larger longitudinal studies incorporating lifestyle, nutritional, and other shared determinants are needed to clarify temporal relationships and identify systemic profiles that may be particularly relevant to periodontal disease.

## 5. Conclusions

In this exploratory cross-sectional study of periodontal patients in a university dental setting, multimorbidity and polypharmacy were common, but increasing numbers of systemic diagnoses and medications were not significantly associated with greater periodontal disease burden after adjustment for age, sex, and current smoking. These findings suggest that simple counts of diagnoses or medications may not adequately capture the aspects of systemic health relevant to periodontal disease. Nevertheless, the high prevalence of multimorbidity and polypharmacy highlights the clinical importance of comprehensive medical and medication assessment in periodontal care. Future longitudinal studies incorporating specific disease and medication patterns, disease severity and duration, and metabolic, inflammatory, and lifestyle-related factors are needed to better characterize the complex relationships between systemic and periodontal health.

## Figures and Tables

**Figure 1:**
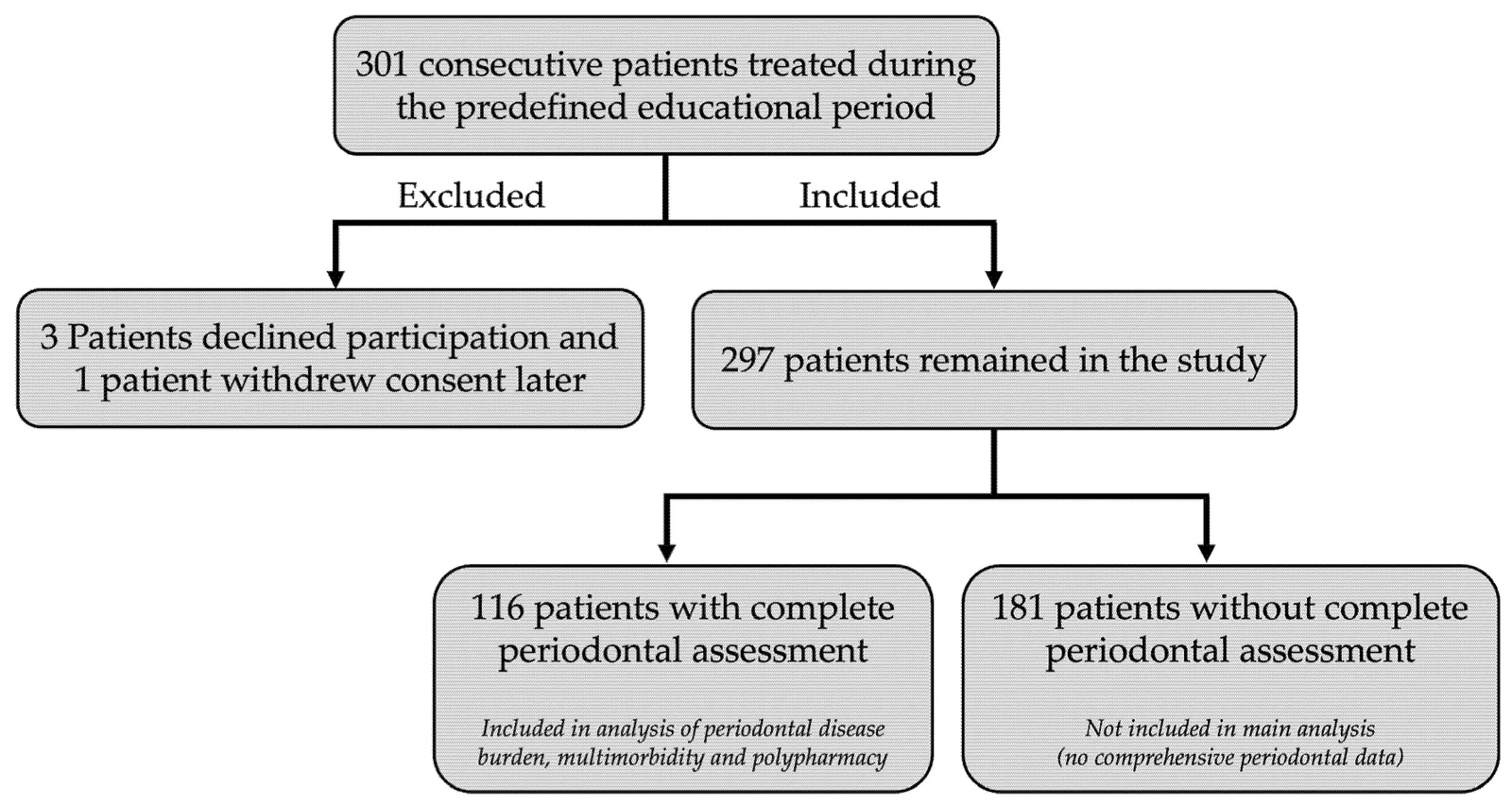
Flowchart of Patient Selection.

**Table 1:** Characteristics of the study population.

Characteristic	Study Population (*n* = 116)
Demographic and behavioral characteristics	
Age, years	63.1 ± 11.2
Female sex	61 (52.6)
Current smoking	30 (25.9)
Systemic disease burden	
≥1 documented systemic condition	85 (73.3)
Number of systemic diagnoses, median (IQR)	2 (0–3.75)
Multimorbidity (≥2 systemic conditions)	66 (56.9)
Medication burden	
Number of medications, median (IQR)	2 (0–4)
Polypharmacy (≥5 medications)	28 (24.1)
Number of ATC medication groups, median (IQR)	1.5 (0–3)
Periodontal characteristics	
Teeth and implants present	22.1 ± 5.0
Mean PPD, mm	3.02 ± 0.58
Mean CAL, mm	3.53 ± 0.86
BOP, %	21.2 (9.5–36.3)
PCR, %	51.8 (38.2–70.2)
Sites with PPD ≥ 4 mm, %	24.0 (12.8–35.9)
Sites with PPD ≥ 6 mm, %	2.5 (0.7–7.4)

Notes: Data are presented as mean ± standard deviation, median (interquartile range), or *n* (%), as appropriate. ATC, Anatomical Therapeutic Chemical classification; CAL, clinical attachment level; PPD, probing pocket depth; BOP, bleeding on probing; PCR, plaque control record; IQR, interquartile range.

**Table 2:** Multivariable associations of systemic disease and medication burden with periodontal parameters.

A. Systemic Disease Burden						
**Predictor**	**Mean CAL, mm β (95% CI)**	*p*-Value	Sites with PPD ≥ 6 mm, % β (95% CI)	*p*-Value	BOP, % β (95% CI)	*p*-Value
Age, per year	0.019 (0.003 to 0.034)	0.022 *	0.069 (−0.084 to 0.221)	0.373	−0.303 (−0.703 to 0.097)	0.137
Sex [female]	−0.179 (−0.328 to −0.029)	0.019 *	−0.418 (−1.845 to 1.010)	0.563	4.255 (0.503 to 8.008)	0.027 *
Smoking [non-current smoker]	−0.327 (−0.521 to −0.133)	0.001 *	−3.376 (−5.215 to −1.537)	<0.001 *	3.557 (−1.277 to 8.391)	0.148
Number of individual diagnoses, per diagnosis	0.026 (−0.040 to 0.092)	0.441	0.382 (−0.255 to 1.019)	0.237	0.285 (−1.389 to 1.959)	0.737
Model R^2^	0.160		0.130		0.068	
Adjusted R^2^	0.129		0.099		0.034	
B. Medication burden						
Predictor	Mean CAL, mm β (95% CI)	*p*-value	Sites with PPD ≥ 6 mm, % β (95% CI)	*p*-value	BOP, % β (95% CI)	*p*-value
Age, per year	0.019 (0.003 to 0.036)	0.023 *	0.072 (−0.088 to 0.232)	0.374	−0.173 (−0.590 to 0.245)	0.414
Sex [female]	−0.181 (−0.331 to −0.032)	0.018 *	−0.442 (−1.875 to 0.991)	0.542	4.007 (0.273 to 7.741)	0.036 *
Smoking [non-current smoker]	−0.328 (−0.524 to −0.131)	0.001 *	−3.359 (−5.223 to −1.495)	<0.001 *	3.003 (−1.853 to 7.860)	0.223
Number of individual medications, per medication	0.011 (−0.048 to 0.070)	0.710	0.222 (−0.347 to 0.792)	0.441	−0.852 (−2.336 to 0.631)	0.257
Model R^2^	0.156		0.124		0.078	
Adjusted R^2^	0.126		0.092		0.045	

Note: Values are unstandardized parameter estimates (β) with 95% confidence intervals from separate multivariable linear regression models. Models in Panel A included age, sex, current smoking, and the number of individual diagnoses; models in Panel B included age, sex, current smoking, and the number of individual medications. Current smoking status was coded as a binary variable (yes/no), and smoking-status data were available for all participants in the analytical cohort; * indicates statistical significance (*p* < 0.05)

**Table 3:** Distribution of major ICD-10 and ATC categories in the study population.

Category	*n* (%)
ICD-10 diagnosis chapters	
Diseases of the circulatory system (IX)	62 (53.4)
Endocrine, nutritional and metabolic diseases (IV)	38 (32.8)
Diseases of the musculoskeletal system and connective tissue (XIII)	19 (16.4)
Diseases of the respiratory system (X)	15 (12.9)
Diseases of the digestive system (XI)	11 (9.5)
ATC medication groups	
Cardiovascular system (C)	50 (43.1)
Blood and blood-forming organs (B)	34 (29.3)
Alimentary tract and metabolism (A)	31 (26.7)
Systemic hormonal preparations, excl. sex hormones and insulins (H)	24 (20.7)
Nervous system (N)	19 (16.4)
Musculoskeletal system (M)	16 (13.8)

**Table 4:** Multivariable associations of selected systemic disease and medication categories with periodontal parameters.

Exposure	Mean CAL, Parameter Estimate (95% CI)	*p*-Value	Sites with PPD ≥ 6 mm, %, Parameter Estimate (95% CI)	*p*-Value	BOP, %, Parameter Estimate (95% CI)	*p*-Value
ICD-10 IV—Endocrine, nutritional and metabolic diseases	−0.048 (−0.212 to 0.116)	0.564	−0.351 (−1.937 to 1.235)	0.662	−1.209 (−5.351 to 2.933)	0.564
ICD-10 IX—Diseases of the circulatory system	−0.028 (−0.206 to 0.151)	0.759	−0.354 (−2.066 to 1.358)	0.683	+2.763 (−1.684 to 7.210)	0.221
ATC A—Alimentary tract and metabolism	−0.094 (−0.266 to 0.077)	0.278	−1.383 (−3.029 to 0.263)	0.099	−0.966 (−5.317 to 3.385)	0.661
ATC C—Cardiovascular system	−0.018 (−0.192 to 0.155)	0.834	−0.411 (−2.081 to 1.259)	0.627	+0.103 (−4.265 to 4.471)	0.963

Note: Values are the parameter estimates and 95% confidence intervals as provided by the multivariable linear regression models in JMP. Models were adjusted for age, sex, and current smoking. For the binary ICD-10 variables, the reported parameter corresponds to level 0; for the binary ATC variables, it corresponds to the “No” level, according to the JMP model coding. CAL, clinical attachment level; PPD, probing pocket depth; BOP, bleeding on probing.

## Data Availability

The data that support the findings of this study are available from the corresponding author, MMH, upon reasonable request.

## Supplementary Materials

The following supporting information can be downloaded at: https://www.mdpi.com/article/10.3390/biomedicines14102153/s1. Supplementary: Table S1: Sensitivity analyses using categorical definitions of multimorbidity and polypharmacy; Supplementary Table S2: Most frequently documented individual systemic diagnoses in the study cohort (*n* = 116); Supplementary Table S3: Most frequently documented medication classes in the study cohort (*n* = 116).

## Abbreviations

The following abbreviations are used in this manuscript:

ACE	Angiotensin-converting enzyme
ATC	Anatomical Therapeutic Chemical Classification System
BOP	Bleeding on probing
CAL	Clinical attachment level
CI	Confidence interval
HbA1c	Glycated hemoglobin (hemoglobin A1c)
ICD-10	International Statistical Classification of Diseases and Related Health Problems, 10th Revision
IQR	Interquartile range
PCR	Plaque control record
PPD	Probing pocket depth
R^2^	Coefficient of determination
SD	Standard deviation
STROBE	Strengthening the Reporting of Observational Studies in Epidemiology
β	Unstandardized regression coefficient
