# Supplementary material for: Multimorbidity, Polypharmacy, and Periodontal Disease Burden in a University Dental Setting: A Cross-Sectional Study
Source: Biomedicines. 2026 Sep 23;14(10):2153. doi: 10.3390/biomedicines14102153 (PMC13701409; doi:10.3390/biomedicines14102153)
Supplement: Supplementary file 1 [file biomedicines-14-02153-s001.zip › biomedicines-4561081-supplementary.pdf]

Supplementary Material

Supplementary Table S1: Sensitivity analyses using categorical definitions of multimorbidity and polypharmacy

Exposure	Outcome	Adjusted β (95% CI)	p-value	R^2
Multimorbidity (≥2 conditions)	Mean CAL, mm	+0.112 (−0.216 to +0.439)	0.500	0.159
	Sites with PPD ≥6 mm, %	+0.871 (−2.31 to +4.05)	0.585	0.122
	BOP, %	−4.243 (−12.44 to +3.96)	0.307	0.076
Polypharmacy (≥5 medications)	Mean CAL, mm	+0.091 (−0.269 to +0.452)	0.617	0.157
	Sites with PPD ≥6 mm, %	+1.688 (−1.77 to +5.15)	0.336	0.126
	BOP, %	−2.764 (−11.83 to +6.31)	0.547	0.070

Note: Values are unstandardized regression coefficients (β) with 95% confidence intervals. β represents the adjusted difference in the periodontal outcome for patients meeting the respective definition compared with those not meeting the definition. All models were adjusted for age, sex, and current smoking. Multimorbidity was defined as ≥2 documented systemic conditions and polypharmacy as ≥5 medications. CAL, clinical attachment level; PPD, probing pocket depth; BOP, bleeding on probing.

Supplementary Table S2: Most frequently documented individual systemic diagnoses in the study cohort (N = 116)

Individual systemic diagnosis	n	%
Hypertension	52	44.8
Type 2 diabetes mellitus	16	13.8
Hypothyroidism	15	12.9
Asthma	11	9.5
Osteoporosis	9	7.8
Heartburn	9	7.8
Rheumatic disease	7	6.0
Coagulation disorder	6	5.2
History of myocardial infarction	6	5.2
Hypotension	6	5.2

Note: Percentages are based on the total study cohort (N = 116). Individual patients could have more than one systemic diagnosis; therefore, percentages do not sum to 100%.

Supplementary Table S3: Most frequently documented medication classes in the study cohort (N = 116)

Medication class	n	%
Antiplatelet agents	18	15.5
Lipid-lowering agents	18	15.5
Thyroid hormones	16	13.8
ACE inhibitors	13	11.2
Diuretics	11	9.5
Beta-blockers	8	6.9
Antidepressants	7	6.0
Oral antidiabetic agents	7	6.0
Anticoagulants	5	4.3

Note: Percentages are based on the total study cohort (N = 116). Individual patients could use more than one medication class; therefore, percentages do not sum to 100%.
